# Robotic sleeve lobectomy with four arms for lung cancer centrally located in the right lower lobe: a case report

**DOI:** 10.1186/s13019-017-0675-4

**Published:** 2017-11-29

**Authors:** Min Seop Jo, Do Yeon Kim, Jin Yong Jeong, Geun Dong Lee

**Affiliations:** 1 Department of Thoracic and Cardiovascular Surgery, St. Vincent’s hospital, College of Medicine, The Catholic University of Korea, Seoul, South Korea; 2 Department of Thoracic and Cardiovascular Surgery, Incheon St. Mary’s Hospital, College of Medicine, The Catholic University of Korea, 56 Dongsu-ro, Bupyeong-gu, Incheon, 21431 Republic of Korea; 3 Department of Thoracic Surgery, Asan Medical Center, Ulsan University College of Medicine, Seoul, Republic of Korea

**Keywords:** Lung cancer, Lobectomy, Robotics

## Abstract

Sleeve lobectomy can preserve healthy lung parenchyma in centrally located lung cancer surgery. Video-assisted thoracoscopic surgery (VATS) lobectomy has been shown to have better results for postoperative complications than thoracotomy lobectomy. However, its limitations in visualization of operative field and handling of instruments restrain surgeons performing sleeve lobectomy. Robotic surgery has several advantages, including magnified 3-dimensional vision and angulation of the robot arm that can provide better circumstances for sleeve lobectomy than VATS. However, robotic sleeve lobectomy has been rarely reported. Here, we describe our experience of performing robotic sleeve lobectomy using four arms for lung cancer centrally located in the right lower lobe.

Dear Sir,

With great interest, we have read the report by Zhao et al. presenting the case of robotic sleeve lobectomy using three arms for lung cancer located at the left lower lobe and projected into the lobe bronchus [1]. Robotic surgery has several advantages, including magnified 3-dimensional vision, angulation of the robot arm, scaled motion, hand tremor filtration, and intuitive movement [2]. It provides better circumstances for sleeve lobectomy than video-assisted thoracoscopic surgery (VATS). Here, we would like to share our experience of performing robotic sleeve right lower lobectomy using four arms for centrally located lung cancer.

A 60-year-old male presented with cough, sputum and dyspnea for a week. Chest computed tomography scans revealed an endobronchial mass in the orifice of the right lower lobe bronchus with necrotizing pneumonia. Bronchoscopic study showed an irregular round mass obstructing the orifice of the right lower lobe bronchus on the distal portion of the right intermediate bronchus without involving the middle lobe bronchi (Fig. 1a). Squamous cell carcinoma was diagnosed by pathology.

**Fig. 1:**
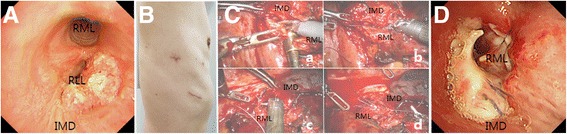
**a** Preoperative bronchoscopy showing a tumor mass obstructing the orifice of the right lower lobe bronchus. **b** Four-arm ports (camera and arm-1, −2, −3) and assistant incisions wounds (arm-3 port incision wound is not shown here). **c** Robotic bronchial anastomosis using four arms. Transected bronchi were trimmed (*a*), a single row of interrupted sutures were started the farthest from the camera (*b*), and the rest of the sutures were completed (*c* and *d*). (**d**) Postoperative bronchoscopy revealing no signs of anastomotic leak or stricture. IMD: intermediate bronchus; RLL: right lower lobe bronchus; RML: right middle lobe bronchus

The patient underwent robotic right lower lobe sleeve lobectomy after being placed on the table in the left decubitus position. He was under endotracheal general anesthesia using a double lumen tube. Robotic thoracic surgery was performed using a da Vinci Si surgical system (Intuitive surgical Inc., Sunnyvale, CA, USA) and the 4-arm robotic technique reported by Cerfolio et al. [3] with modifications. Briefly, two 8-mm port incisions for arm 1 and arm 2 were made in the 6th intercostal space along the anterior axillary line and near the tip of the scapula. A 5-mm port incision for arm 3 was placed in the 6th intercostal space between the tip of the scapula and the spine. A 12-mm camera port incision was placed between arm 1 and 2 ports. Instead of placing an assistant port as described by Cerfolio et al., a 3.5-cm access assistant incision was made in the 8th intercostal space to form a triangle with the camera port and arm 1 port (Fig. 1b).

Robotic arms were docked with approach from the left side of the patient head at a 15° angle. Mobilization and transection of the right lower pulmonary vein and arteries of lower lobe were done using 45-mm staplers (ECR45W, White, ECHELON FLEX™ Powered ENDOPATH® Stapler, Ethicon, Inc., Cincinnati, Ohio, United States). Bronchi were dissected and transected on the right middle lobe and intermediate bronchi with scissors (monopolar curved scissors). The specimen was removed by access incision. Frozen pathologic examination confirmed negative resected margins. Transected intermediate and middle lobe bronchi were trimmed for end-to-end anastomosis. A single row of interrupted sutures with 4–0 Vicryl was started from the cartilaginous portion the farthest from the camera. One-half of sutures were performed followed by lateral traction sutures of 2–0 Vicryl on either side of both bronchi. After that, the rest of sutures were completed (Fig. 1c). Systemic mediastinal lymph node dissection was performed. The bronchial anastomosis was covered with a parietal pleural flap. Bronchoscopy performed on the 7th postoperative day revealed no signs of anastomotic leak or stricture (Fig. 1d). The patient has been followed for 5 months without surgical complications.

VATS has several limitations for sleeve lobectomy. Using a traditional endoscopic needle holder is inconvenient for suture or knot tying. In addition, articulation of manual endoscopic suture is limited. VATS view is especially limited in suturing membranous portion [4]. On the other hand, robotic surgery has the advantages, including magnified 3-dimensional vision and angulation of the robot arm that make sleeve lobectomy more convenient than VATS [2, 4]. Lin et al. performed robotic sleeve lobectomy with three arms in the six patients and demonstrated the feasibility of robotic surgery in complex lung cancer surgeries [4].

Use of a fourth arm in robotic thoracic surgery provides additional advantages compared to the three-arm technique. It reduces the requirement of instrument change by the assistant. It permits retraction of the lung and tissues directly by the surgeon himself. It allows exposure and tensioning of the operating field exactly as the surgeon prefers. In addition, it allows the assistant to use access incision to insert ancillary instruments [5]. Therefore, there is virtue for the fourth arm in robotic surgery. In our case, we also used the fourth arm, a 5-mm thoracic grasper, to retract the lung for sleeve resection of the right lower lobe bronchus and make the resected bronchi more stable for anastomosis.

In summary, a patient presented lung cancer centrally located in the right lower lobe bronchus. We performed robotic surgery with modification of 4-arm robotic technique. It provides additional advantages for sleeve lobectomy compared to 3-arm robotic technique.

## Abbreviations

VATS: Video-assisted thoracoscopic surgery
